# Collateral sensitivity and cross-resistance in six species of bacteria exposed to six classes of antibiotics

**DOI:** 10.1128/spectrum.00983-25

**Published:** 2025-06-20

**Authors:** Xinyu Wang, Luyuan Nong, Gioia Schaar, Belinda Koenders, Martijs Jonker, Wim de Leeuw, Benno H. ter Kuile

**Affiliations:** 1 Biology and Microbial Food Safety, Swammerdam Institute for Life Sciences, University of Amsterdam 1234, Amsterdam, the Netherlands; 2 RNA Biology and Applied Bioinformatics, Swammerdam Institute for Life Sciences, University of Amsterdam 1234, Amsterdam, the Netherlands; Universita degli Studi dell'Insubria, Varese, Italy

**Keywords:** *fusA *mutation, collateral sensitivity, cross-resistance, convergent evolution, kanamycin resistance

## Abstract

*De novo* resistance can develop in bacteria as a result of exposure to sublethal concentrations of antibiotics. Once the strain has become resistant to an initial antibiotic, this can cause cross-resistance or collateral sensitivity to a second antimicrobial. Specific collateral sensitivity is rarely conserved across species because the mechanisms triggered in different microorganisms to resist antibiotics are often different. In this study, we explored which collateral sensitivity or cross-resistance networks are present in six species of bacteria with induced *de novo* resistance. These six species were induced to become resistant to amoxicillin/cefepime, enrofloxacin, kanamycin, tetracycline, erythromycin, and chloramphenicol (T. Pulingam, T. Parumasivam, A. M. Gazzali, A. M. Sulaiman, J. Y. Chee, et al., Eur J Pharm Sci 170:106103, 2022, https://doi.org/10.1016/j.ejps.2021.106103). In this study, the collateral sensitivity and the cross-resistance networks were evaluated by measuring the increase or decrease of MIC of 13 antibiotics that are often used in the clinic. Collateral sensitivity for kanamycin occurred in five species made resistant to chloramphenicol and tetracycline and for β-lactams in five species made resistant to kanamycin. Genetic analysis revealed that *fusA* consistently mutated in five bacterial species that exhibited *de novo* resistance to kanamycin, suggesting that mutations in domain IV of *fusA* are the primary contributors to the consistent CS phenotype observed in these species. Based on considerations of resistance, a treatment protocol starting with chloramphenicol/tetracycline, followed by kanamycin, and ending with amoxicillin may eliminate bacteria that have developed resistance against the initial treatment.

**IMPORTANCE:**

Collateral sensitivity and cross-resistance influence the outcome of antimicrobial infection treatments. To evaluate the potential effects of these phenomena, collateral sensitivity and the cross-resistance networks were documented by measuring the MIC of six species of bacteria with induced resistance against 13 antibiotics. These effects are indeed, in some cases, clinically relevant. One example was further explored: collateral sensitivity for kanamycin in five species made resistant to chloramphenicol and tetracycline and for β-lactams in five species made resistant to kanamycin. In this case, genetic analysis revealed that *fusA* consistently mutated in the five bacterial species that exhibited *de novo* resistance to kanamycin. The observed collateral sensitivity can be explained by these mutations.

## INTRODUCTION

Antibiotic-resistant bacterial infections have become a serious public health concern across the world (1). When bacteria initially become resistant to one antibiotic, this may increase or decrease the susceptibility to other antibiotics compared to naïve wild-type strains, defined as collateral sensitivity (CS) and cross-resistance (CR), respectively (2). Cross-resistance can occur due to multi-target mechanisms, such as efflux pumps (3–5). Persistence of CR hampers clearing pathogens that have become resistant to the affected antimicrobials. Inversely, collateral sensitivity might be used to advance treatment options. In that case, alternative treatments could be developed based on CS to eliminate bacteria resistant to one antibiotic using specifically designed treatments with other compounds (6, 7). Antibiotic combinations that take advantage of CS could be designed to be more effective than the individual antibiotics alone, thereby reducing the development of resistance (6, 8).

Both CS and CR have been described in a variety of bacterial species, and using CS is regarded as a viable technique to combat antibiotic resistance (9). Resistance to ciprofloxacin, a fluoroquinolone, increased susceptibility to the aminoglycoside tobramycin and to the β-lactam aztreonam in *Pseudomonas aeruginosa* (6, 10). Similarly, tobramycin resistance increased the susceptibility for phosphomimic acid but not for ciprofloxacin (11). Collateral sensitivity networks have been identified in *Escherichia coli*. For example, aminoglycoside resistance is associated with reduced effectiveness of the AcrAB efflux system that pumps out other antibiotics due to a reduction of the electrochemical proton gradient (12). Various bacteria employ different mechanisms to resist a specific antibiotic, resulting in a similar level of resistance to that antibiotic while exhibiting different CS phenotypes to other antibiotics. The CS in multiple species could in principle be used to increase the effectiveness of antibiotic treatments in hospitals (9), provided that the CS networks have been documented.

This study aimed at identifying the CS and CR networks in six species of bacteria made resistant against 6 antibiotics each and 13 in total. In a preceding study, *Staphylococcus aureus*, *Enterococcus faecalis*, *Bacillus subtilis*, *Yersinia enterocolitica*, *Salmonella enterica*, and *Acinetobacter pittii* were induced to evolve *de novo* resistance against six different classes of antibiotics: β-lactam, fluoroquinolones, aminoglycosides, tetracyclines, macrolides, and chloramphenicols (13). No species was made resistant against more than six antibiotics, avoiding compounds against which a species was inherently resistant. In the present study, the strains thus obtained were used to address the following questions: to which extent do collateral sensitivity and cross-resistance occur after the development of *de novo* resistance? Can patterns of CS be observed across species? Are the appearances of CS and CR consistent enough to function as a foundation for the development of strategies to prevent antimicrobial resistance? The availability of 72 strains representing six species made resistant using the exact same methodology to six antibiotics, in duplicate, allowed us to answer these questions with a degree of completeness that could not be achieved otherwise.

## RESULTS

**Fig 1:**
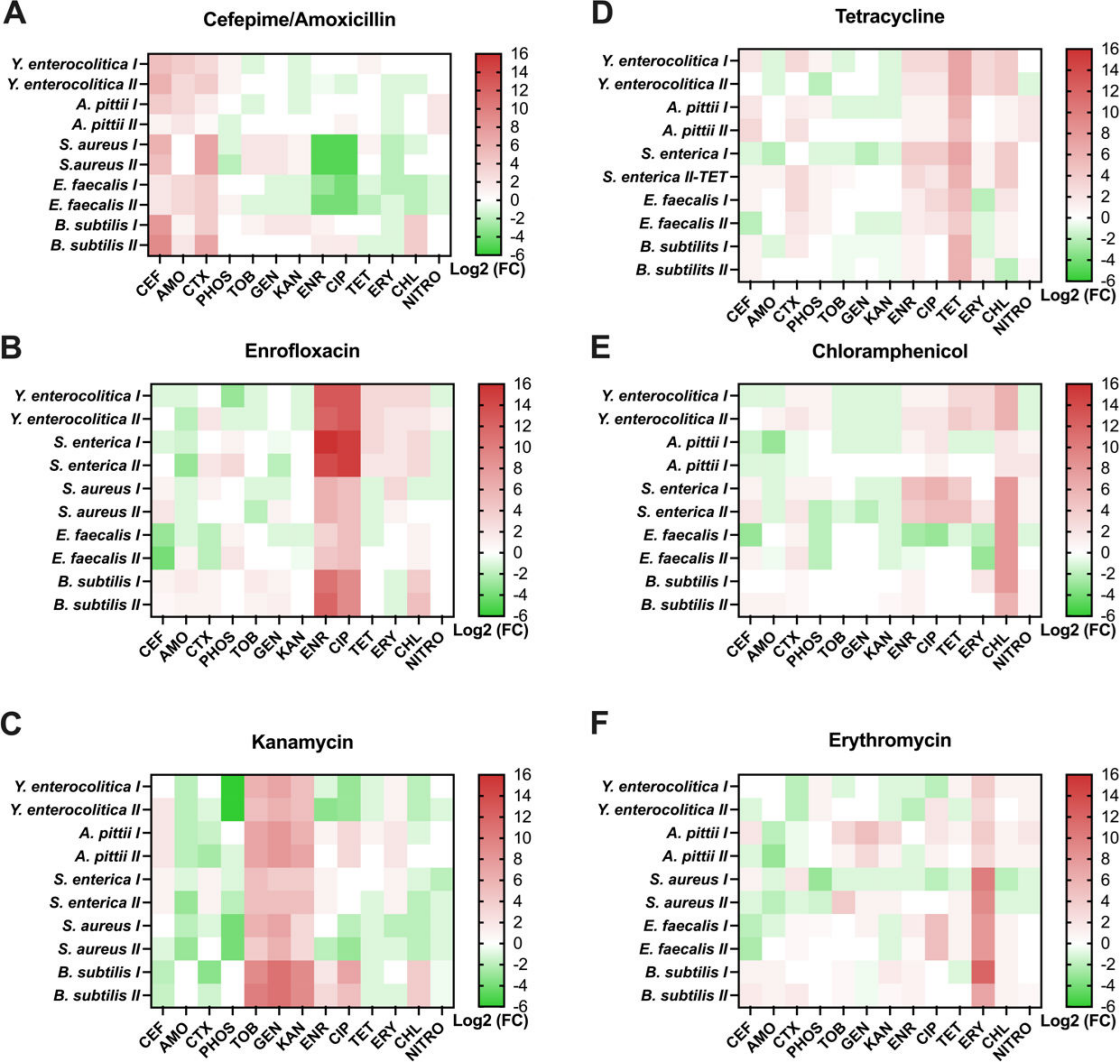
Duplicates of each strain were exposed to antibiotics, and the MIC of the resulting strains was measured using two technical replicates. Calculations were made using the average of the four values thus obtained. Visualization of collateral sensitivity and cross-resistance of strains made resistant to (**A**) amoxicillin (AMO)/cefepime (CEF), (**B**) enrofloxacin (ENR), (**C**) kanamycin (KAN), (**D**) tetracycline (TET), (**E**) chloramphenicol (CHL), and (**F**) erythromycin (ERY). The intensity of red represents the degree of cross-resistance, and the intensity of green represents the degree of collateral sensitivity based on the log2 of the fold change (FC) in MIC values of Table 1.

For a study of the DNA mutations associated with *de novo* antimicrobial resistance, the six bacterial species mentioned above were exposed to stepwise increasing concentrations of six antibiotics (13). For this study, the final resistance was measured against 13 antibiotics of the strains thus obtained in order to identify patterns in the occurrence of CS and CR. Because of inherent resistance, not all combinations of drugs and species were relevant (Fig. 1). CS and CR are visualized for six bacterial species made *de novo* resistant against at least one of the six classes mentioned in the introduction. At first view, cross-resistance seems to occur more often than collateral sensitivity. This conclusion is incorrect because, in these heatmaps, the original antibiotic against which resistance was developed is also included, sometimes accompanied by chemically closely related compounds. Cross-resistance is mostly limited to antibiotics from the same class, while collateral sensitivity primarily occurs between chemically unrelated antibiotics (Fig. 1). Strong CS occurred between fluoroquinolone resistance and induced cefepime resistance in *S. aureus* and *E. faecalis*. Cefepime and amoxicillin induce more often CS than the other antimicrobials tested. The aminoglycosides used in this study show mild CS with several other antimicrobials across species. The strains of *S. enterica* exposed to amoxicillin, *A. pittii* to enrofloxacin, *E. faecalis* to kanamycin, *S. aureus* to tetracycline, *S. enterica* to erythromycin, and *S. aureus* to chloramphenicol all barely developed resistance, with a maximum fourfold increase in minimal inhibitory concentration (MIC). Consequently, they did not develop noticeable CS and CR.

**TABLE 1:** List of antibiotics used in the collateral sensitivity testing

Antibiotic	Abbreviation	Class	Target
Cefepime	CEF	β-Lactam	Cell wall
Amoxicillin	AMO	β-Lactam	Cell wall
Cefotaxime	CTX	β-Lactam	Cell wall
Kanamycin	KAN	Aminoglycoside	Protein synthesis, 30S
Gentamycin	GEN	Aminoglycoside	Protein synthesis, 30S
Tobramycin	TOB	Aminoglycoside	Protein synthesis, 30S
Enrofloxacin	ENR	Fluoroquinolone	DNA gyrase
Ciprofloxacin	CIP	Fluoroquinolone	DNA gyrase
Tetracycline	TET	Tetracycline	Protein synthesis, 30S
Erythromycin	ERY	Macrolide	Protein synthesis, 50S
Chloramphenicol	CHL	Amphenicol	Protein synthesis, 50S
Phosphomimic	PHOS	Fosfomycin	Cell wall biogenesis
Nitrofurantoin	NITRO	Dihydrofolate reductase inhibitor	Folic acid metabolism

One of the most compelling findings is the conserved CS observed in five strains resistant to tetracycline/chloramphenicol, kanamycin, and amoxicillin/cefepime. Five species of bacteria resistant to tetracycline/chloramphenicol did not show any CR to aminoglycosides. Instead, they exhibited slight or clear CS to aminoglycoside antibiotics. Additionally, the strains resistant to kanamycin displayed CR to gentamicin and tobramycin while maintaining CS to amoxicillin and phosphonomycin. Based on the outcome of the whole-genome sequencing (WGS) analysis of these strains (13), the convergence of kanamycin resistance across five species can primarily be attributed to a common mutation in the *fusA* gene, especially in domain IV, which drives most kanamycin resistance (13). The *fusA* gene codes for the elongation factor G in *E. coli*, which is important for bacterial protein synthesis and is associated with drug resistance in *Clostridium difficile* (14). This gene was mutated in all replicates during the development of kanamycin resistance (Table 2). The number of times that an alanine positioned in the 500s was replaced is striking (seven times). Most of the time, the new amino acid was valine. The reverse only happened once, at position 86. Around the location of alanine replacement, glycine (5×) and phenylalanine (6×) were almost as often replaced. Glycine was most often replaced by cysteine and phenylalanine by leucine, isoleucine, or valine. Otherwise, only two serine residues were replaced: a single threonine and one valine. All the amino acid replacements can be the result of a single nucleotide change. However, the overall effect on kanamycin resistance is still striking, so the selective advantages must have been considerable.

**TABLE 2:** Mutations of *fusA* in six species as a result of induced kanamycin resistance

Species	Gene	Mutation type	Time point	Amino acid change	Allele frequency
*S. enterica-1*	*fusA*	Missense_variant	1 | 2	Ala608Val	1 | 1
	*fusA*	Missense_variant	2	Phe601Val	1
*S. enterica*-2	*fusA*	Missense_variant	1 | 2	Thr668Ala	0.25 | 0.555
	*fusA*	Missense_variant	1	Phe605Val	0.64
	*fusA*	Missense_variant	2	Ala493Gly	0.361
	*fusA*	Missense_variant	2	Ser589Ala	0.561
*Y. enterocolitica*-1	*fusA*	Missense_variant	1 | 2	Ala590Val	0.628 | 1
	*fusA*	Missense_variant	1 | 2	Phe603leu	0.307 | 1
*Y. enterocolitica*-2	*fusA*	Missense_variant	1	Phe603Leu	0.565
	*fusA*	Missense_variant	2	Ala590Val	0.923
*A. pittii*-1	*fusA*	Missense_variant	1	Gly148Cys	0.949
	*fusA*	Missense_variant	2	Phe535lle	1
*A. pittii*-2	*fusA*	Missense_variant	1	Ala595Val	1
	*fusA*	Missense_variant	2	Phe535lle	1
*E. faecalis-*1	*fusA*	Missense_variant	1 | 2	Gly665Cys	0.948 | 1
*E. faecalis-*2	*fusA*	Missense_variant	1	Ala539Glu	0.427
	*fusA*	Missense_variant	1 | 2	Gly665Cys	0.2085 | 1
*B. subtilis*-1	*fusA*	Missense_variant	1 | 2	Val86Ala	0.993 | 1
*B. subtilis*-2	*fusA*	Missense_variant	2	Gly551Cys	1
*S. aureus*-1	*fusA*	Missense_variant	1	Gly507Ser	1
	*fusA*	Frame shift	2	Ser481dup	0.980
*S. aureus*-2	*fusA*	Missense_variant	2	Ala580Val	0.85

## DISCUSSION

The effects of CS have primarily been investigated in the framework of resistance evolution of single laboratory species (12, 14, 15). The investigation of the convergence of CS phenotypes in six *de novo* resistant strains against 13 antibiotics in this study focused on the mechanisms underlying CS. One can assume that the cellular adjustments and/or DNA mutations that result in resistance to one antibiotic make the cell more vulnerable to a second antimicrobial with a different mode of action. In this study, CS occurred for various combinations of bacterial species and two antibiotics: the initial that induced resistance and the second that either showed CS or not (drugs/bug). Even when CS is reproducible for specific drugs/bug combinations, it is usually not conserved after growth for some time in the absence of the initial antibiotic evolution (16–18). This suggests that both CS and CR can be caused by adaptation at the level of cellular mechanisms, rather than DNA mutations. The trade-off between functions that cells make, such as maintaining pH or salt balance against pumping out an antibiotic, can cause either CR or CS. If an induced efflux pump is effective against two antibiotics with different modes of action, the result will be cross-resistance (19). If the efflux pump is not effective against the second drug but still uses energy that is then not available for combatting the second antibiotic, CS will be the outcome.

Questions remain regarding the convergence of CS phenotypes across species. Recent studies examined single strains with mutational backgrounds (11, 20, 21), clinical isolates from a single species (9, 15), and different *Enterococcus faecium, Staphylococcus aureus, Klebsiella pneumoniae, Acinetobacter baumannii, Pseudomonas aeruginosa*, and *Enterobacter* spp. (ESKAPE) pathogens (9). Since the evolutionary trajectory is limited by the genomic context, the CS phenotypes are rarely consistent between various species, leading to treatment failure in the clinic (11, 21). However, the target mutations in the node of the evolution trajectory, which have a positive epistasis with the other mutations, are rarely absent in the set of acquired mutations, thereby shaping a robust resistance phenotype (6, 22).

**Fig 2:**
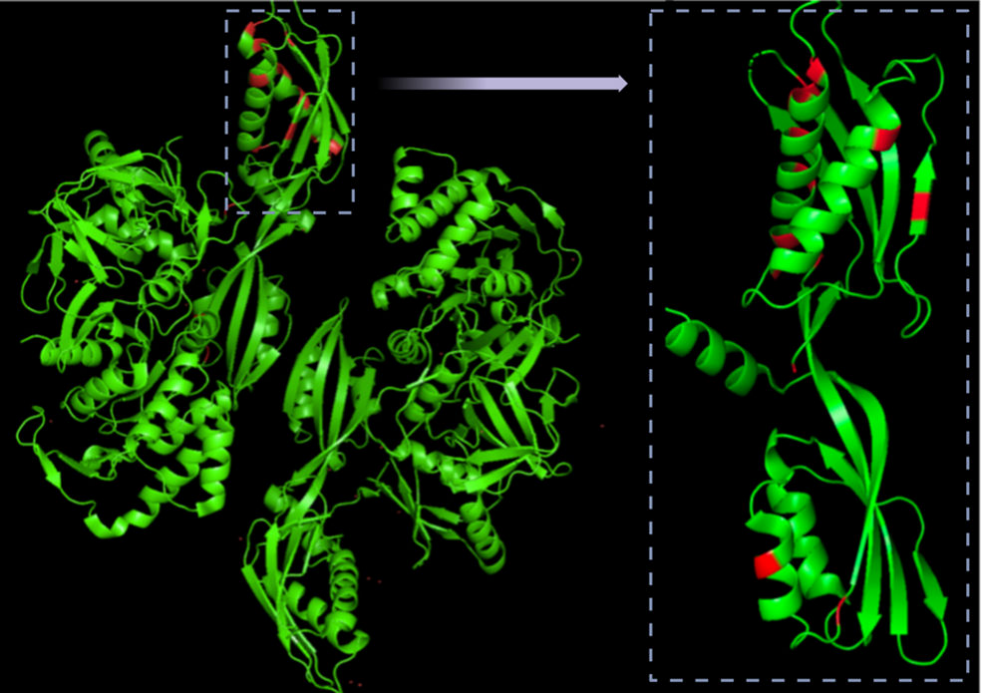
Structural effects of the mutations occurring in the FusA protein of *S. aureus* PDB3ZZT (23). The *fusA* mutations were identified in six bacterial species through whole-genome sequencing (13). All amino acid substitutions in FusA for each strain were subsequently mapped onto the FusA protein structure (*S. aureus*, PDB: 3ZZT). These mutations predominantly occurred in a hotspot region within domain IV of the FusA protein, which is highlighted in red.

A discrete CS phenotype is observed across species, while CR is only conserved within strains resistant to the same class of antibiotics. Even when strains develop high levels of *de novo* antibiotic resistance, the species do not use the same mechanisms. WGS analysis of newly fluoroquinolone-resistant strains revealed that only *gyrA* and *parC* are conserved across all six species, resulting in varying CS profiles among them (13). The variation in resistance mechanisms is also confirmed by the observations of various mutational pathways in the resistance evolution of the ESKAPE species (9), revealing a discrete CS phenotype in these pathogens. However, the convergent kanamycin resistance development in five species observed in the present study is characterized by the high frequency of *fusA* mutations. Elongation factor G is a protein with five domains that bind to the 70S ribosome (23) (Fig. 2). During the translocation steps, domain IV interacts with the ribosomal A site, facilitating the movement of mRNA and tRNA from the A site to the P site on the ribosome (24, 25). After kanamycin resistance evolution, most *fusA* mutations occurred in domain IV, which may alter the affinity of kanamycin for the ribosomal protein, potentially reducing the drug’s effectiveness.

The mutations that occurred at domain IV in *fusA* result in the same level of resistance to tobramycin and gentamicin in five species, indicating that the *fusA* mutations might be the main contributors to aminoglycoside resistance. *FusA* mutations were also acquired in tobramycin evolution in *A. baumannii* and *P. aeruginosa* in biofilm and planktonic conditions, implying that the mutations are essential for bacteria to survive in different niches (26). The strains resistant to chloramphenicol and tetracycline did not induce cross-resistance to aminoglycoside antibiotics. Tetracycline and chloramphenicol are bacteriostatic antibiotics and induce less *de novo* resistance than bactericidal compounds (19). This resistance is caused more by cellular adjustments at the expression level than by DNA mutations (20). As a result, they cause more CS, because the adjustments that increase tolerance to bacteriostatic antibiotics apparently increase the sensitivity to bacteriostatic antimicrobials. In addition, the mutations in *fusA* exert a pleiotropic effect (27), which reduces the reaction to nutritional stress sensors (ppGpp), decreasing heme levels, thereby enhancing oxidative stress and DNA damage. As a result, it increases the susceptibility to β-lactam antibiotics (27), which explains the collateral sensitivity of β-lactam observed in kanamycin-resistant strains (26). Based on the CS findings, we propose a follow-up treatment line using chloramphenicol/tetracycline, kanamycin, and amoxicillin/phosphonomycin to minimize the effects of induced resistance.

In conclusion, exposure to bacteriostatic antibiotics increases their sensitivity to aminoglycosides, which in turn makes the bacteria more susceptible to β-lactam antibiotics. Therefore, a theoretical sequence of antimicrobials for persistent infection that develops resistance during treatment can be imagined. Based solely on considerations of preventing the development of resistance, one would arrive at the following sequence: start with a bacteriostatic, follow up with an aminoglycoside, and clear the infection with a β-lactam. Whether this is relevant for the clinic or veterinary practice needs to be investigated.

## MATERIALS AND METHODS

### Medium, bacterial strains, and drugs

The resistant strains were derived from naive wild types by exposure to stepwise increasing sublethal concentrations. The wild-type strains were *Salmonella enterica* DSM 9221, *Staphylococcus aureus* DSM 110565, *Enterococcus faecalis* ATCC 4707711, *Acinetobacter pittii* ATCC 33305, *Bacillus subtilis* B168, and *Yersinia enterocolitica* ATCC 9610. The strains acquired resistance against the drugs listed in Table 2. The antibiotics were chosen so that each species was exposed to a member of the six classes of antibiotics, but antimicrobials against which inherent resistance existed were avoided. The MIC measurements (28, 29) and overnight cultures were performed in tryptic soy broth (TSB) medium. Strains were stored at −80°C and streaked onto TSB agar medium for use in overnight cultures. The list of antibiotics used can be found in Table 2. Antibiotic solutions were made from powder stocks (Sigma) and filter sterilized. The antibiotic solutions were kept at −20°C, or −80°C for phosphomimic and cefepime, for up to 90 days. Every 5 days, a new batch of amoxicillin was prepared. The antibiotic solutions were stored at <4°C for a maximum of 4 days.

### *De novo* resistance acquisition

The six species of bacteria developed *de novo* resistance against six antibiotics in the framework of a study on the accompanying genetic mutations (13). The resulting strains were used in this study. To develop *de novo* resistance acquisition, the wild-type bacteria were initially grown at one-eighth and a quarter of their MIC for that specific antibiotic. The cell cultures were grown in TSB medium with a starting optical density at 600 nm (OD_600_) of 0.1 for 24 hours at 200 rpm at 37°C for *B. subtilis*, *S. aureus*, *E. faecalis*, and *S. enterica* and at 30°C for *Y. enterocolitica* and *A. pittii*. Strains were grown at two antibiotic concentrations, the lower one being half of the higher. If the OD_600_ of the high concentration strains was 75% or more compared to the optical density (OD) of the low-level incubation, the experiment was continued with a twofold increase of both antibiotic concentrations. If the OD of the highly exposed was below 75% of the lower level exposure, the same concentrations were maintained until the strain adapted to growth at the higher concentration. The *de novo* resistance evolution was executed with biological duplicates and terminated when no further increase could be observed, usually after 20–40 transfers. Throughout the resistance evolution experiments, the MICs were measured on both replicates.

### Minimal inhibitory concentration measurement

The MIC was measured by following growth at stepwise, by a factor of 2, increasing concentrations of antibiotics in 96-well plates (Thermo Fisher Scientific) (28, 29). Overnight cultures were prepared in TSB medium and incubated at 200 rpm at 37°C for *B. subtilis*, *S. aureus*, *E. faecalis*, and *S. enterica* and at 30°C for *Y. enterocolitica* and *A. pittii*. The MICs were measured on the duplicate evolving or evolved strains with a minimum of two technical replicates for each. The bacteria were added per well with an OD of 0.05. Afterward, the plates were incubated at 200 rpm for 23 hours at 30°C or 37°C, respectively. Plates were shaken in the plate reader for 2 minutes and measured afterward at an absorbance of 600 nm.

### Analysis of collateral sensitivity and cross-resistance

The six strains that evolved high levels of resistance to six antibiotics by the end of the evolution experiment were selected for CS and CR detection by MIC measurements. Heatmaps and graph bar collections were created using the software GraphPad Prism version 9. To calculate the degree of collateral sensitivity or cross-resistance, the following formula was used: log_2_ [MIC_(res)_ / MIC_(WT)_]. A negative number represents collateral sensitivity, and a positive outcome represents cross-resistance compared to the wild type.

### DNA isolation and fusA mutation analysis

A total of 144 samples from evolved populations, collected at the middle and final time points of their evolutionary trajectory, along with 6 biological control samples and 6 wild-type samples, were subjected to genomic DNA sequencing (13). The sequencing details are provided in reference 13. While the outcome of that study describes the molecular consequences of induced resistance, the present study focuses on the *fusA* mutations. All of the *fusA* mutations found in the six species of bacteria were aligned to the FusA protein in *S. aureus* PDB3ZZT (23).

## Data Availability

The whole-genome sequencing and assembly data are deposited in NCBI SRA under BioProject no. PRJNA1194003. All other original data can be requested from the corresponding author.

## SUPPLEMENTAL MATERIAL

Table S1 spectrum.00983-25-s0001.docx Log2 fold change of the measured MICs.

ASM does not own the copyrights to Supplemental Material that may be linked to, or accessed through, an article. The authors have granted ASM a non-exclusive, world-wide license to publish the Supplemental Material files. Please contact the corresponding author directly for reuse.
