# Supplementary material for: Collateral sensitivity and cross-resistance in six species of bacteria exposed to six classes of antibiotics
Source: Microbiol Spectr. 2025 Jun 20;13(8):e00983-25. doi: 10.1128/spectrum.00983-25 (PMC12323328; doi:10.1128/spectrum.00983-25)
Supplement: Table S1 — Log2 fold change of the measured MICs. [file spectrum.00983-25-s0001.docx]

|  | CEFE | AMOX | CTX | PHOS | TOB | GEN | KAN | ENR | CIP | TET | ERY | CHL | NITRO |
| --- | --- | --- | --- | --- | --- | --- | --- | --- | --- | --- | --- | --- | --- |
| Y. enterocolitica I-AMOX | 5 | 4 | 3 | 1 | -1 | 0 | -1 | 0 | 0 | 1 | 0 | 0 | 0 |
| Y. enterocolitica II-AMOX | 6 | 3 | 4 | 1 | 0 | 0 | -1 | 0,42 | -1 | 0 | -1 | -1 | 0 |
| A. pittii I-AMOX | 4 | 3 | 1,42 | 0 | -1 | 0 | -1 | 0 | 0 | 0 | -1 | 0 | 2 |
| A. pittii II-AMOX | 1 | 2 | 0,42 | -1 | 0 | 0 | 0 | 0 | 1 | 0 | -1 | 0 | 2 |
| S. aureus I-CEFE | 6 | 0 | 7 | -1 | 2 | 2 | 1 | -5 | -5 | 1 | -2 | -1 | 0 |
| S.aureus II-CEFE | 5 | 0 | 7 | -2 | 2 | 2 | 1 | -5 | -5 | 0 | -2 | 0 | 0 |
| E. faecalis I-CEFE | 2 | 3 | 4 | 0 | 0 | -1 | -1 | -3 | -4 | -1 | -2 | -2 | -1 |
| E. faecalis II-CEFE | 2 | 3 | 4 | 1 | -1 | -1 | -1 | -4 | -4 | -2 | -1 | -2 | -1 |
| B.subtilis I-CEFE | 8 | 1 | 4 | 0 | 1 | 2 | 2 | 0,94 | 0 | 0 | -1 | 4 | 0 |
| B.subtilis II-CEFE | 9 | 2 | 7 | 0 | 0 | 0 | 0 | 1,53 | 1,58 | -1 | -1 | 4 | 0 |
| S. aureus I-ENRO | 1 | -1 | 1 | 0 | -1 | -1 | 0 | 6 | 5 | -1 | 3 | -1 | -1 |
| S. aureus II-ENRO | 2 | -1 | 0 | 0 | -2 | 1 | 0 | 6 | 5 | -1 | 1 | 0 | 0 |
| S. enterica I-ENRO | -1 | 1,40 | 0 | 1 | 0 | 0,42 | 0 | 16 | 15 | 3 | 2 | 3 | -1 |
| S. enterica II-ENRO | 0 | -3 | 2 | 3 | 0 | -2 | 0 | 14 | 15 | 2 | 2 | 3 | -1 |
| Y. enterocolitica I-ENRO | -1 | -1 | 0 | -3 | -1 | 0 | -1 | 13 | 13 | 3 | 3 | 3 | -1 |
| Y. enterocolitica II-ENRO | 0 | -2 | 2 | -1 | -1 | 0 | -1 | 12 | 13 | 3 | 2 | 2 | 1 |
| E. faecalis I-ENRO | -3 | -1 | -2 | 1 | 0 | -1 | -1 | 3,42 | 5 | -1 | 0 | 1 | 0 |
| E. faecalis II-ENRO | -4 | 1 | -2 | 2 | 0 | 0 | -0,42 | 4,42 | 5 | 0 | 0 | 1 | 0 |
| B. subtilis I-ENRO | 1 | 1,58 | 1 | 0 | 1,58 | 1 | 0 | 10,94 | 9 | 0 | -1 | 4 | 0 |
| B. subtilis II-ENRO | 0,58 | 1 | 1 | 0 | 1 | 1,32 | 0 | 11,94 | 9 | 0 | -1 | 5 | 0 |
| S. aureus I-KAN | 0 | -2 | -1 | -4 | 6 | 7 | 3 | 0 | -2 | -1 | -2 | -2 | -1 |
| S. aureus II-KAN | -1 | -3 | 0 | -4 | 4 | 6 | 3 | -2 | -3 | -1 | -1 | -2 | -1 |
| S. enterica I-KAN | 1 | -1 | 1 | -1 | 5 | 4 | 4 | 1 | 0 | 0 | 1 | -1 | -2 |
| S. enterica II-KAN | 1 | -3 | 1 | -2 | 5 | 5 | 5 | 1 | 0 | -1 | 1 | -2 | -1 |
| Y. enterocolitica I-KAN | 0 | -2 | 0 | -6 | 6 | 7 | 5 | -1 | -3 | -1 | 1 | -2 | 0 |
| Y. enterocolitica II-KAN | 2 | -2 | -1 | -6 | 5 | 6 | 5 | 3,32 | -3 | -1 | 1 | -2 | -1 |
| A. pittii I-KAN | 2 | -2 | 1,58 | 0 | 7 | 8 | 6 | 1 | 3 | 1 | 2 | -1 | 0 |
| A. pittii II-KAN | 2 | -2 | 2,58 | -1 | 7 | 8 | 7 | 0 | 3 | 0 | 2 | 0 | 0 |
| B. subtilis I-KAN | -2 | 0 | 3,41 | 0 | 9 | 11 | 9 | 1,94 | 7 | -1 | 0 | 4 | 0,42 |
| B. subtilis II-KAN | 1,68 | 0 | 1,42 | 0 | 10 | 11 | 9 | 2,94 | 5 | -1 | -1 | 4 | 0,42 |
| Y. enterocolitica I-TET | 2 | -1 | 3 | 1 | -1 | 0 | -1 | 3 | 3 | 7 | 3 | 4 | 0 |
| Y. enterocolitica II-TET | 0 | -1 | 0 | -2 | 0 | 0 | -1 | 2 | 2 | 7 | 3 | 4 | -1 |
| A. pittii I-TET | 2 | 0 | 1,41 | 1 | -1 | -1 | -1 | 1 | 1 | 6 | 0 | 1 | 2 |
| A. pittii II-TET | 3 | 0 | 2,42 | 0 | 0 | 0 | 0 | 1 | 1 | 5 | 0 | 1 | 2 |
| S. enterica I-TET | -1 | -2 | 0 | -1 | -1 | -2 | -1 | 4 | 4 | 7 | 1 | 4 | 0 |
| S. enterica II-TET | 1 | 1 | 3 | 1 | 0,58 | 0 | 0 | 3 | 2 | 6 | 1 | 4 | 0 |
| E. faecalis I-TET | 1 | 0 | 3 | 1 | 0 | 0 | 0 | 1,42 | 3 | 5 | -2 | 2 | 0 |
| E. faecalis II-TET | -2 | 0 | 2 | 1 | 0 | -1 | -1 | 1,42 | 2 | 4 | -1 | 1 | 0 |
| B. subtilits I-TET | 1 | -1 | 1,58 | 0 | 0,42 | 0,42 | 0,42 | 0,94 | 0 | 6 | -1 | 1 | 0 |
| B. subtilits II-TET | 1 | 0 | 0 | 0 | 0,42 | 0 | 0,42 | 0,94 | 0 | 6 | 1 | -2 | 0,58 |
| Y. enterocolitica I-CHL | -1 | -1 | 1 | 1 | -1 | -1 | -1 | 1 | 1 | 3 | 3 | 6 | -1 |
| Y. enterocolitica II-CHL | 0 | 1 | 2 | 1 | -1 | -1 | -1 | 2 | 2 | 4 | 3 | 6 | -1 |
| A. pittii I-CHL | 1,42 | -3 | 0,58 | 0 | -1 | -1 | -1 | 1 | 2 | -1 | -1 | 2 | 1 |
| A. pittii I-CHL | -1 | -1 | 0,58 | 0 | 0 | 0 | 0 | 0 | 1 | 0 | 0 | 2 | 2 |
| S. enterica I-CHL | 1 | -1 | 1 | 1 | 0 | -1 | -1 | 5 | 6 | 4 | 0 | 8 | 1 |
| S. enterica II-CHL | 2 | -1 | 2 | -2 | -1 | -2 | -1 | 4 | 5 | 5 | 2 | 8 | 1 |
| E. faecalis I-CHL | -3 | 0 | 1 | -2 | 0 | 0 | -1 | 2,58 | -3 | -1 | -2 | 8 | -1 |
| E. faecalis II-CHL | 1 | 0,42 | 2 | -2 | 0 | 0 | -1 | 0,58 | 0 | 0 | -3 | 8 | 0 |
| B. subtilis I-CHL | 0 | 0 | 0,58 | 0 | 0 | 0 | 0 | 0,94 | 0 | 0 | 2 | 8 | 0,58 |
| B. subtilis II-CHL | 1 | 1 | 0,58 | 0 | 0 | 0 | 0,58 | 0,94 | 0 | 0 | 0 | 6 | 0,58 |
| Y. enterocolitica I-ERY | 0 | 0 | -2 | 1 | -1 | 0 | -1 | -1 | -2 | 1 | 4 | 1 | 1 |
| Y. enterocolitica II-ERY | -1 | 0 | -2 | 1 | 0 | 0 | -1 | -2 | 2 | -1 | 3 | 0 | 1 |
| A. pittii I-ERY | 2 | -2 | 0,58 | 0 | 3 | 5 | 3 | 0 | 2 | 1 | 4 | 1 | 2 |
| A. pittii II-ERY | -1 | -3 | 0,58 | 0 | 1 | 3 | 1 | -1 | 1 | 0 | 3 | 1 | 1 |
| S. aureus I-ERY | 1 | -1 | 2 | -3 | -1 | -1 | -1 | -1 | -2 | -1 | 10 | -2 | -1 |
| S. aureus II-ERY | -1 | -2 | -1 | 1,41 | 4 | 1 | 1 | 0 | 0 | 1 | 9 | -1 | -1 |
| E. faecalis I-ERY | -2 | -1 | 0,58 | 0,46 | 0 | 1 | -1 | 1,42 | 5 | 1 | 8 | 1 | 0 |
| E. faecalis II-ERY | 2,42 | 0 | 0,58 | 0 | 0 | 0 | -1 | 0,42 | 5 | 1 | 8 | 1 | 0 |
| B. subtilis I-ERY | 1 | 1 | 0 | 0 | 0,58 | -0,42 | 1,58 | 0,94 | 0 | -1 | 12 | 1 | 0 |
| B. subtilis II-ERY | 1,58 | 1 | 1,58 | 0 | 0,58 | 0,42 | 0,58 | 0,94 | 0 | 0 | 7 | 1 | 0,58 |
